# Cardiac CT Angiography in Diagnosing Patent Foramen Ovale: A Study on Patients with Suspected Patent Foramen Ovale-Associated Stroke

**DOI:** 10.3390/jcdd13020075

**Published:** 2026-02-02

**Authors:** Lijie Sun, Chong Zheng, Zhenxing Fan, Jing Gao, Zhi Liu, Jin Si, Keling Xiao, Ming Yi, Haoyu Zhang, Jinghao Sun, Yijin Liu, Yang Hua, Yingqi Xing, Jie Lu, Jing Li

**Affiliations:** 1 Department of Geriatrics, Xuanwu Hospital Capital Medical University, National Clinical Research Center for Geriatric Diseases, Beijing 100053, China; sunlijie@xwhosp.org (L.S.);; 2 Department of Radiology and Nuclear Medicine, Xuanwu Hospital Capital Medical University, Beijing 100053, China; 3 Department of Cardiology, Xuanwu Hospital Capital Medical University, Beijing 100053, China; 4 Department of Emergency, Xuanwu Hospital Capital Medical University, Beijing 100053, China; liuzhi@xwhosp.org; 5 Department of Vascular Ultrasound, Xuanwu Hospital Capital Medical University, Beijing 100053, China

**Keywords:** computed tomographic angiography, cryptogenic stroke, patent foramen ovale, transesophageal echocardiography, contrast transcranial Doppler

## Abstract

Background: Cardiac computed tomographic angiography (CTA) detects patent foramen ovale (PFO) with variable accuracy. This study investigated factors affecting CTA detectability for PFO in patients with suspected PFO-associated stroke. Methods: Consecutive patients with cryptogenic stroke and positive findings on contrast transcranial Doppler (cTCD) examinations were enrolled between November 2020 and April 2023 in this retrospective study. Each participant underwent transesophageal echocardiography (TEE) and cardiac CTA. Patients with confirmed PFO on TEE were categorized into two groups based on CTA detectability: the CTA-positive group (PFO identified by CTA) and the CTA-negative group (PFO missed by CTA). Univariate and multivariate logistic regression analyses were performed to identify predictors of CTA false-negative results. Results: Among 108 patients (mean age 46.7 ± 14.9 years, 47.2% male), the prevalence of PFO by TEE was 94.4% (102/108). Compared to TEE, cardiac CTA had a sensitivity of 70% (95% CI 61–79%), a specificity of 100% (95% CI 54–100%), a positive predictive value of 100% (95% CI 95–100%), and a negative predictive value of 16% (95% CI 6–32%). Among patients with PFO confirmed by TEE (*n* = 102), the incidence of moderate to large right-to-left shunts (RLS) was significantly higher in the CTA-positive group than in the CTA-negative (77.5% vs. 22.5%, *p* < 0.001). After adjusting for confounders, patients with moderate to large shunts showed a significantly lower likelihood of a CTA false-negative result compared to those with small shunts (OR 0.113, 95% CI 0.035–0.365, *p* < 0.001). In patients with moderate to large RLS, the sensitivity of cardiac CTA for diagnosing PFO increased to 90.16% (95% CI 82.69–97.64%). Conclusions: Cardiac CTA could be an effective complementary modality for selected patients with suspected PFO-associated stroke. Its diagnostic performance appears more reliable for identifying PFO in patients with moderate-to-large RLS than in those with small RLS.

## 1. Introduction

Patent foramen ovale (PFO)-associated stroke is a recently proposed classification of ischemic stroke, occurring in patients with PFO after excluding other potential causes of stroke [1]. Epidemiological studies indicate that PFO-associated stroke accounts for 5% of ischemic strokes, with this proportion exceeding 10% in adults aged 18 to 60 years. Consequently, screening PFO should be mandatory when evaluating cryptogenic stroke [2]. Screening imaging modalities include contrast transcranial Doppler (cTCD), transthoracic echocardiography (TTE), transesophageal echocardiography (TEE), and agitated saline contrast echocardiography [3]. According to guidelines and expert consensus, TEE is regarded as the “gold standard” for diagnosing PFO and conducting anatomical evaluations prior to closure [4]. Nevertheless, it is not considered a mandatory examination. As a semi-invasive technique, TEE may induce discomfort during the procedure, which compromises patient cooperation. It is also associated, although rarely, with potential life-threatening complications [5]. Alternative modalities, such as cTCD and TTE, also have certain limitations. cTCD can reliably detect right-to-left shunt (RLS) but cannot localize the anatomical origin of the shunt [6]. TTE has limited sensitivity (50–73.3%) due to signal attenuation from thoracic structures and false positives from intrapulmonary shunt [7,8]. Therefore, optimizing the PFO diagnostic algorithm remains a clinical priority for screening causes of ischemic stroke.

Cardiac computed tomography angiography (CTA), with its high temporal and spatial resolution, enables comprehensive visualization of intracardiac pathologies including thrombi, valvular anomalies, cardiac neoplasms, and complex aortic atherosclerotic plaques [9,10]. It demonstrates considerable utility in etiological screening for acute ischemic stroke [11]. Notably, cardiac CTA facilitates non-invasive diagnosis of PFO through dynamic visualization of contrast jets and channel-like appearances (CLA), which represent the anatomical tunnel between atrial chambers [12]. But its diagnostic performance for PFO detection varies significantly across studies, with sensitivity ranging from 25% to 83% [13,14]. Kim et al. demonstrated that cardiac CTA had a sensitivity of 73.1% (95% CI 64.2–81.0%) and a specificity of 98.4% (95% CI 94.6–99.8%) in a comparative cohort of 152 stroke patients [15]. Conversely, Rinkel et al. reported the sensitivity of cardiac CTA is 25% (95% CI 14–94%) in acute ischemic stroke patients [16]. Diagnostic discrepancies may be attributed to various factors, including populations’ selection bias, individual heterogeneity, and technical parameters [17]. Identifying influencing factors could facilitate the clinical application of cardiac CTA. However, relevant research remains limited.

In this study, we investigated the potential factors affecting the detectability of cardiac CTA in the identification of PFO and to optimize the clinical application of this imaging modality in patients with suspected PFO-associated stroke.

## 2. Materials and Methods

Consecutive patients with suspected PFO-associated stroke admitted to Xuanwu Hospital Capital Medical University were enrolled from November 2020 to April 2023, and each patient underwent both cardiac CTA and TEE during the same hospitalization. No specific treatments were performed between the two examinations. Patients with confirmed PFO on TEE were categorized into two groups based on CTA detectability: the CTA-positive group (PFO identified by CTA) and the CTA-negative group (PFO missed by CTA). Baseline characteristics were compared between the two groups.

### 2.1. Study Population

**Figure 1:**
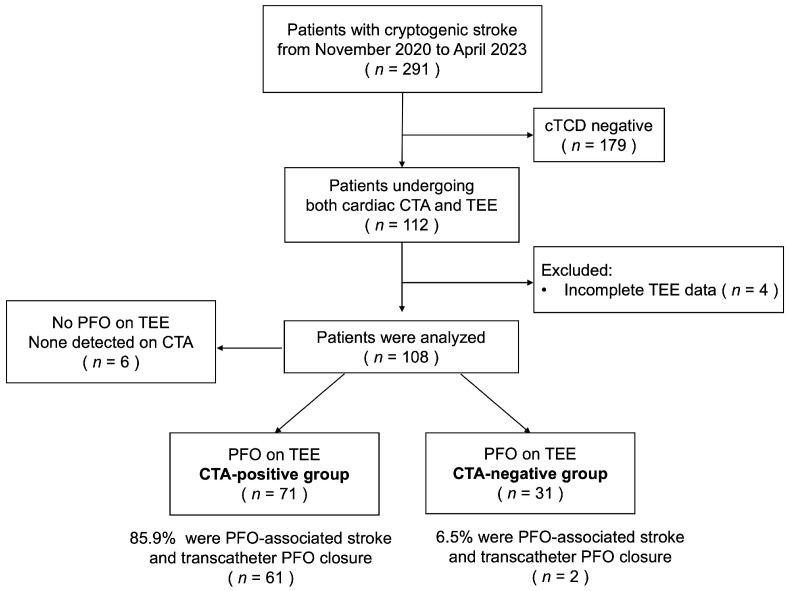
Flowchart of the study population. cTCD, contrast-enhanced transcranial Doppler; TEE, transesophageal echocardiography; CTA, computed tomography angiography.

The study flowchart is presented in Figure 1. All patients presenting with symptoms of ischemic stroke underwent initial evaluation in the Department of Neurology. This comprehensive evaluation included cranial CT or magnetic resonance imaging, carotid and cerebral artery ultrasonography, 24 h or longer cardiac rhythm monitoring, coagulation and immunological function tests, and cTCD examination. Patients lacking an identifiable cause of stroke and exhibiting positive findings on cTCD were considered potential candidates for PFO-associated stroke and were subsequently enrolled in this study. They were referred to the Department of Cardiology for PFO detection using TEE and cardiac CTA, the images of which were independently interpreted by specialized teams led by senior cardiologists.

The study was conducted in accordance with the Declaration of Helsinki and received approval from the Ethics Committee of Xuanwu Hospital, Capital Medical University (NO. 2023048). Written informed consent was obtained from all patients.

### 2.2. Cardiac CTA Examination and Image Reconstruction

All CTA examinations were performed using a third-generation dual-source CT scanner (SOMATOM Force; Siemens Healthineers, Forchheim, Germany). The patients underwent scanning with a retrospective electrocardiograph-gated technique in a breath-holding state.

Contrast medium was administered into the right antecubital vein at a flow rate of 5.0 mL/s using a high-pressure syringe, followed by 25 mL of normal saline at the same flow rate. Automated bolus-tracking technology was used, with the region of interest positioned at the level of the descending aorta. Scanning was initiated automatically 5 s after the trigger threshold surpassed 80 Hounsfield units (HU). The scan covered an area from the tracheal bifurcation to approximately 1 cm below the diaphragmatic surface of the heart. The acquisition parameters were set as follows: tube voltage at 120 kV, tube current at 300 mA, pitch factor at 1.375, field of view at 40 cm, and slice thickness at 0.625 mm. During scanning, images with a reconstruction interval of 5 mm were automatically generated by the CT system for routine review. Total scan time ranged from 6.0 to 9.0 s.

**Figure 2:**
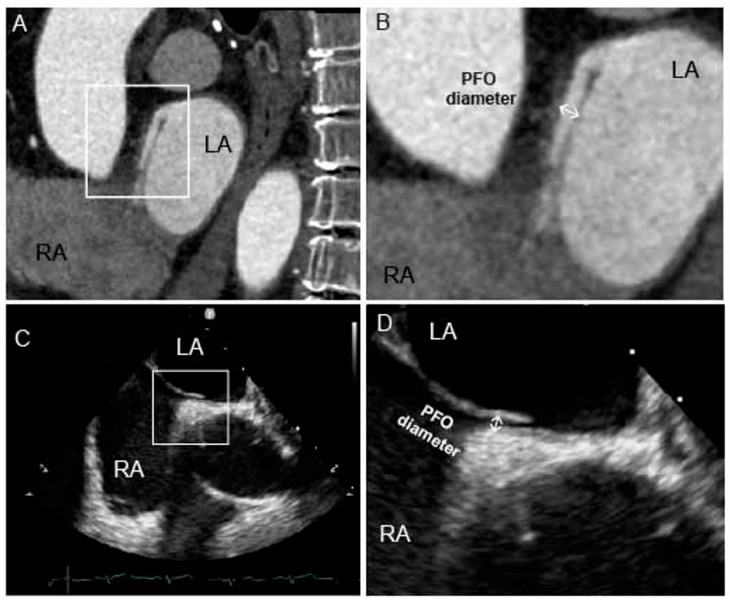
PFO diagnosed on TEE and cardiac CTA. (**A**) Oblique sagittal reformation CT images demonstrate the channel-like appearance of interatrial septum and contrast agent jets from LA to RA toward inferior vena cava. (**B**) PFO diameter in cardiac CTA was measured in its middle level from flap valve to superior interatrial groove. (**C**) TEE shows incomplete closure of the interatrial septum. (**D**) PFO diameter in TEE was measured as maximum distance between the septum primum and septum secundum during continuous scanning of the interatrial septum. LA, left atrial; RA, right atrial; PFO, patent foramen ovale; TEE, transesophageal echocardiography; CTA, computer tomography angiography.

Following acquisition, the raw CTA data were transferred to a Siemens post-processing workstation (Syngo.via VB40, Siemens Healthineers, Forchheim, Germany) for reconstruction. The images were reconstructed with a slice thickness of 1 mm and a reconstruction interval of 1 mm for further analysis, using mid-diastolic and end-systole phase. Axial images exhibiting optimal clarity and absence of motion artifacts were selected by consensus for multiplanar reconstruction of the fossa ovalis region. The criteria for detecting PFO in cardiac CTA include the presence of a CLA with a contrast jet (Figure 2A). CLA was defined as a continuous contrast-filled tract between the septum primum and septum secundum, and contrast jet was defined as a linear column of contrast agent extending from the left atrium into the right atrium during the diastolic phase. To ensure diagnostic reliability, this tract was required to be visible on at least two consecutive slices in the axial view and confirmed on sagittal or coronal views. Using the four-chamber view as an anatomical reference, sagittal–oblique planes perpendicular to the interatrial septum were generated to visualize the PFO tunnel. The PFO diameter was measured at the midpoint of the tunnel (Figure 2B). The analysis of cardiac CTA images was performed by two radiologists, both of whom were blinded to the TEE and cTCD results. Any discrepancies in interpretation were resolved by consensus.

### 2.3. TEE Protocol

TEE was performed using a Philips CX50 (Philips Healthcare, Andover, MA, USA) equipped with a 4–7 MHz multiplane transducer. All patients underwent a comprehensive TEE examination, which included standardized views of the interatrial septum, two-dimensional imaging, color Doppler flow assessment, and agitated saline contrast testing. The echocardiographic images were independently analyzed by two researchers. A PFO was identified as an incompletely fused interatrial channel, as shown in Figure 2C. The diameter of the PFO was measured as the maximum distance between the septum primum and septum secundum during continuous scanning of the interatrial septum (Figure 2D). The agitated saline contrast test was performed with a mixture of 9 mL of saline with 1 mL of air and a drop of the patient’s blood, which was administered through the right antecubital vein. To ensure consistency across examinations (cTCD, TEE, and CTA), the same injection site was prioritized for each patient, unless compromised venous access required a change. RLS was identified by the presence of microbubbles in the left atrium within three cardiac cycles. The shunts were categorized based on the number of microbubbles: a small shunt was defined as having 1–10 microbubbles; a moderate shunt as having 11–30 microbubbles; and a large shunt as having more than 30 microbubbles.

### 2.4. Contrast-Enhanced Transcranial Doppler (cTCD) Examination

cTCD was performed using a Multi-DopX4 TCD detector (DWL, Solingen, Germany) with a 2 MHz probe, which facilitated monitoring of the middle cerebral artery through the temporal window. The agitated saline contrast test was carried out as previously described. The agitated saline solution was injected three times at rest and three times during the Valsalva maneuver, which was standardized by instructing patients to exhale forcefully into a manometer until achieving and maintaining a pressure of 50–60 mmHg for at least 5–7 s. Based on the appearance of microbubbles in the cTCD spectrum, shunts were categorized into three grades: a small shunt with 1–10 microbubbles; a moderate shunt with 11–30 microbubbles; and a large shunt with more than 30 microbubbles or when individual microbubbles were indistinguishable within the cTCD spectrum.

### 2.5. Statistical Analysis

Using TEE as the reference standard, the diagnostic performance of cardiac CTA for PFO detection were evaluated. A Shapiro–Wilk test was used to verify whether continuous variables met the normal distribution. Continuous variables with normal distribution were presented as mean ± standard deviation, and non-normal variables were reported as median (interquartile range). Group comparisons were made using Fisher’s exact test, χ2 test, or Student’s t tests, as appropriate. The agreement between TEE and cardiac CTA for measuring the diameter of PFO was calculated using Bland–Altman plots. To identify independent predictors of CTA false-negative results, a multivariate logistic regression analysis was performed. Variable selection was guided by statistical significance (*p* < 0.05 in univariate analysis) and clinical relevance. Specifically, potential confounders known to influence cardiac imaging quality or PFO characteristics were included if they met the statistical threshold. All statistical analyses were performed by using SPSS software (version 26.0, IBM SPSS, Chicago, IL, USA). Statistical significance was assumed at a level of *p* ≤ 0.05.

## 3. Results

### 3.1. Diagnostic Accuracy of Cardiac CTA in Detecting PFO

A total of 108 patients were included in the study, with a mean age of 46.7 ± 16.9 years, and 47.2% of them were male. Of these, 102 patients (94%) were diagnosed with a PFO through TEE, while 6 patients (5.6%) were not diagnosed with PFO. Cardiac CTA accurately identified PFO in 71 (69.6%) of all patients with PFO, while it failed to visualize PFO in 31 (30.4%) patients. Furthermore, cardiac CTA correctly identified all patients without PFO. As demonstrated in Table 1, the sensitivity of cardiac CTA was 70% (95% CI 61–79%), while its specificity was 100% (95% CI 54–100%). The positive and negative predictive values were 100% (95% CI 95–100%) and 16% (95% CI 6–32%), respectively.

### 3.2. Agreement Between Cardiac CTA and TEE in Measuring PFO Diameter

**Figure 3:**
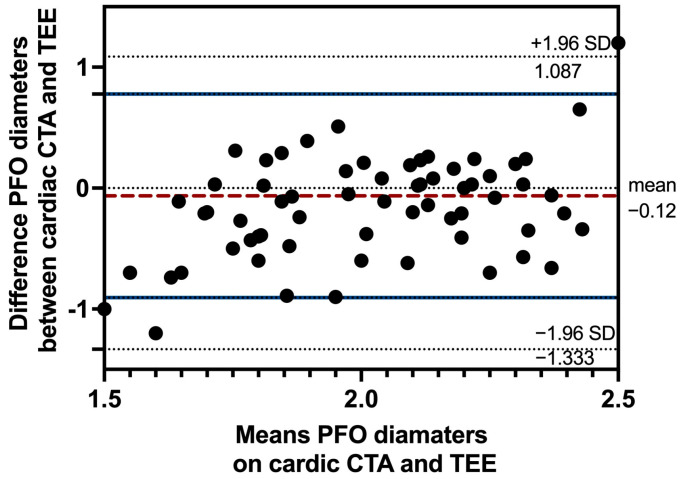
Bland–Altman plots showing the agreement analysis of PFO diameter measured by cardiac CTA and TEE. Bland–Altman plots showing the agreement as determined by cardiac CTA and TEE. The mean difference is 0.12 ± 0.62 mm. The 95% limit of agreement is from −1.33 to 1.09 mm. PFO, patent foramen ovale; TEE, transesophageal echocardiography; CTA, computer tomography angiography.

The diameter of the PFO is a critical anatomical parameter for transcatheter closure in patients with PFO-associated strokes. We analyzed agreement between cardiac CTA and TEE in measuring PFO diameter. This analysis included images from 71 patients with PFO confirmed by both modalities (Figure 2). The average PFO diameter measured by cardiac CTA was 2.03 ± 0.59 mm, compared to 2.15 ± 0.36 mm by TEE. In Figure 3, Bland–Altman plots demonstrated a mean inter-method difference of −0.12 ± 0.62 mm, with 95% limits of agreement ranging from −1.33 mm to 1.09 mm. The distribution of differences showed no systematic bias trend across measurement ranges, with 3/71 (4.2%) data points outside the 95% limits of agreement.

### 3.3. Patient Characteristics

Patients were categorized into two groups based on CTA detectability for PFO detection: CTA-positive group (PFO identified by CTA) and CTA-negative group (PFO missed by CTA). Table 2 presents the baseline characteristics of both groups. Patients in the CTA-positive group were younger (44.0 ± 12.8 vs. 52.9 ± 17.5 years, *p* = 0.005) and had a lower prevalence of chronic disease, including hypertension (25.4% vs. 54.8%, *p* = 0.004) and coronary artery disease (CAD) (1.4% vs. 22.6%, *p* < 0.001), compared with those in the CTA-negative group. The RoPE score in the CTA-positive group was significantly higher than in the CTA-negative group (6.1 ± 1.8 vs. 4.5 ± 2.2 score, *p* < 0.001). The proportion of patients classified as ‘probable’ under the PASCAL classification system was greater in the CTA-positive group than in the CTA-negative group (39.4% vs. 9.7%, *p* < 0.001).

The anatomical characteristics of PFO and the functional shunt between the two groups are shown in Table 3. There were no statistically significant differences in anatomical parameters as measured by TEE between groups, including PFO diameter, atrial septal aneurysm, Eustachian valve and atrial septal defect. Nevertheless, the prevalence of moderate to large RLS was significantly higher in the CTA-positive group compared to the CTA-negative group, as determined by contrast-enhanced TEE (67.6% vs. 25.8%, *p* < 0.001) and cTCD (77.5% vs. 22.5%, *p* < 0.001).

### 3.4. The Factors Influencing the Agreement Between Cardiac CTA and TEE

The factors influencing the agreement between cardiac CTA and TEE were investigated using both univariable and multivariable logistic regression analyses, with results presented in Table 4. After adjusting for age, gender, hypertension, CAD, and LA diameter, moderate to large RLS was significantly associated with a significantly reduced risk of a false-negative CTA result (OR 0.113, 95% CI 0.035–0.365, *p* < 0.001).

**Figure 4:**
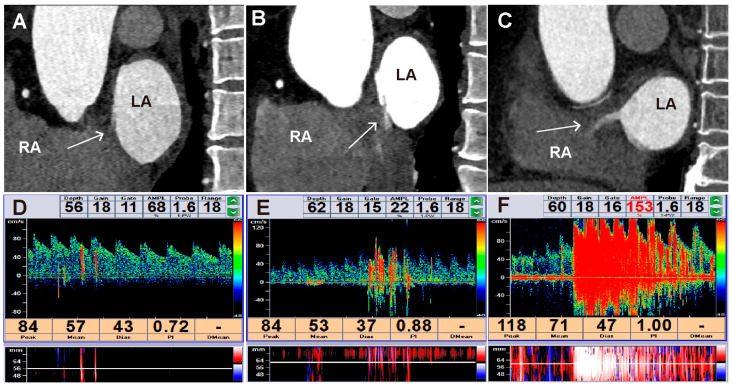
PFO image characteristics in cardiac CTA corresponding to cTCD shunt grades. (**A**) Small shunt in cardiac CTA: maximum jet length ≤ 1 cm (or indiscernible) (arrow). (**B**) Moderate shunt in cardiac CTA: jet length 1–2 cm (arrow). (**C**) Large shunt in cardiac CTA: jet length > 2 cm (arrow). (**D**) Small shunt in cTCD: 1–10 microbubbles. (**E**) Moderate shunt in cTCD: 11–30 microbubbles. (**F**) Large shunt: more than 30 microbubbles or when individual microbubbles were indistinguishable. LA, left atrium; RA, right atrium; CTA, computer tomography angiography; cTCD, contrast-enhanced transcranial Doppler.

Table 5 displays the sensitivity of cardiac CTA in diagnosing PFO among various subgroups. Among the 61 patients with PFO who had moderate to large RLS confirmed by cTCD, cardiac CTA correctly diagnosed PFO in 55 patients, achieving a sensitivity of 90.16% (95% CI 82.69–97.64%). Figure 4 illustrates the different grades of interatrial shunt observed using cardiac CTA and cTCD.

## 4. Discussion

Our findings suggest that cardiac CTA can serve as an effective diagnostic tool for patients with suspected PFO-associated stroke. Its diagnostic performance appears more reliable for identifying PFO in patients with moderate-to-large RLS than in those with small RLS. Given that patients with moderate to large RLS are considered candidates for transcatheter PFO closure, our results also indicate that cardiac CTA can serve as a valuable diagnostic tool in these patients.

Unlike traditional treatments for atherosclerosis-related stroke, transcatheter closure demonstrated superior efficacy over medical therapy in preventing stroke recurrence in patients with PFO-associated stroke [18]. It is crucial to accurately identify PFO in patients with cryptogenic stroke to select the most appropriate treatment strategy [19]. TEE, considered the “gold standard” for PFO diagnosis, has limited applicability due to its semi-invasive procedure, which is often not well tolerated [20]. Although complications are rare, they can occur during the procedure. Furthermore, many primary healthcare facilities often lack TEE equipment, and reliance on TTE combined with agitated saline contrast may lead to underdiagnosis of PFO [21]. TEE is not considered a mandatory examination for screening PFO according to current guidelines [3,4]. Alternative imaging modalities are essential to enhance the diagnostic algorithms for PFO.

Cardiac CTA is a reliable alternative modality for evaluating cardioembolic sources in ischemic stroke, capable of imaging and diagnosing PFO [22,23]. However, its diagnostic performance for PFO detection varies across different studies. Lee et al. reported that the sensitivity and specificity of cardiac CTA were 89.4% and 92.3%, respectively [13], while Kara et al. reported a sensitivity of 53% and a specificity of 75% [24]. These variations may be attributed to differences in the selection of target populations. In cohorts of acute stroke patients, the sensitivity of cardiac CTA for diagnosing PFO was only 25% [16], whereas it increased to 81.8% in patients with ischemic stroke [13]. This study reported 70% sensitivity for PFO detection using cardiac CTA in patients suspected of having PFO-associated strokes. This sensitivity is slightly lower than that reported in studies using dedicated PFO protocols. This discrepancy is likely attributable to the retrospective nature of our study, where the primary indication for cardiac CTA in most patients was the evaluation of coronary artery disease rather than PFO screening. Consequently, a standard coronary CTA protocol was utilized without the Valsalva maneuver, and image acquisition timing was optimized for the coronary arteries rather than the right atrium. Thus, the diagnostic efficacy of cardiac CTA may be influenced by patient heterogeneity and diagnostic criteria. Unlike TEE, which directly visualizes the anatomical details of the primary and secondary septum through ultrasound signals, cardiac CTA utilizes on X-ray tomography technology to identify PFO, mainly through the anatomical features of CLA or contrast jets between the left and right atrium. Miki et al. found that the positive predictive value of “CLA combined with contrast jet” was higher (90.5%) than that of CLA alone (45.7%) for PFO detection [23]. These findings underscore the significant impact of interatrial shunt characteristics on the diagnostic accuracy of cardiac CTA for PFO.

Our results suggest that the degree of the interatrial shunt significantly influences the diagnostic accuracy of cardiac CTA in diagnosing PFO. Left-to-right shunting is common in most patients with PFO and right-to-left shunting [25]. Cardiac CTA relies on the pressure gradient between the left and right atrium to facilitate the shunting of contrast agent, thereby indirectly imaging the shunting. Insufficient pressure or small shunt volumes may lead to insufficient contrast agent shunting signs, potentially resulting in undetected PFOs. In addition, patients with PFO accompanied by moderate to large RLS requires special attention in clinical practice, given their high risk of stroke induced by thrombus passage through the PFO. Patients with a RoPE score exceeding [6] and a large shunt are classified as “probable” according to the PASCAL classification system, suggesting a causal relationship between their strokes and the PFO. These patients are likely to benefit from transcatheter PFO closure [17]. Consequently, the degree of interatrial shunt not only influences the diagnostic accuracy of cardiac CTA for PFO detection but also plays a crucial role in risk stratification and therapeutic decision-making in PFO-associated stroke. Therefore, it is essential to evaluate the degree of right–left shunting by cTCD before cardiac CTA for PFO detection. However, it is important to acknowledge the technical limitations of standard cardiac CTA. In our study, patients performed a standard inspiratory breath-hold solely to minimize respiratory motion artifacts. The absence of a Valsalva maneuver is a critical factor limiting diagnostic sensitivity, particularly for small shunts. Under resting conditions without provocation, the interatrial pressure gradient may be insufficient to open the flap valve of a small PFO, causing it to remain functionally closed and undetectable on static CT images. Future studies implementing technical adjustments could significantly enhance diagnostic accuracy. Specifically, dynamic CTA, which acquires images throughout the cardiac cycle during a Valsalva maneuver, has shown promise in visualizing the transient opening of the PFO flap. Additionally, incorporating delayed-phase imaging may help capture shunts that are not visible during the peak arterial phase.

Evaluating the anatomical structure of PFO and measuring its diameter are crucial for selecting the appropriate occluder devices for transcatheter PFO closure. Saremi et al. reported that the capability of cardiac CTA to measure key anatomical features of the PFO, including the length of the free edge of interatrial septum, the length of the fossa ovalis, and the thickness of the septum secundum [26]. Similarly, Xiong et al. found that cardiac CTA and TEE provided consistent measurements of the PFO tunnel length and the diameter of the left atrial entrance [14]. The present study showed that PFO diameters measured by cardiac CTA showed consistency with the results of the TEE measurement. However, the absence of standardized cardiac CTA protocols for measuring PFO anatomical structures may result in significant discrepancies across different studies, thereby limiting the clinical applicability of this technique.

**Figure 5:**
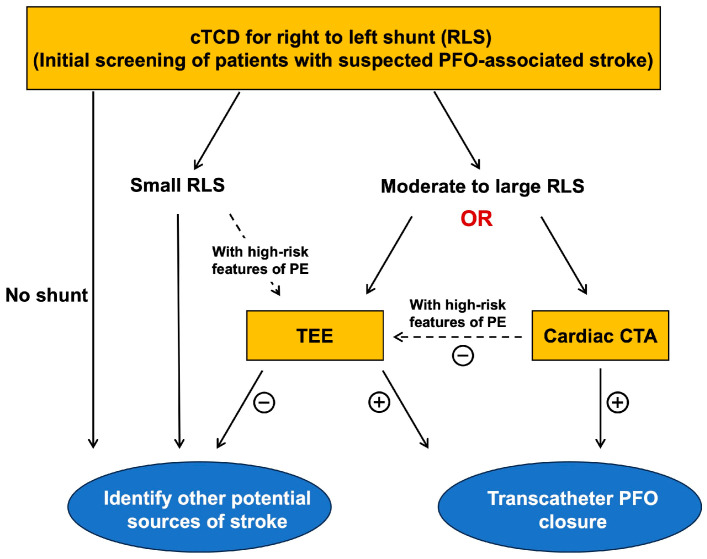
Proposed diagnostic algorithm for PFO-associated stroke. cTCD can be used as the initial screening tool for patients with suspected PFO-associated stroke. Specifically, patients exhibiting moderate-to-large RLS can be evaluated using either TEE or cardiac CTA. PFO, patent foramen ovale; TEE, transesophageal echocardiography; CTA, computer tomography angiography; cTCD, contrast-enhanced transcranial Doppler; RLS, right- to-left shunt; PE, paradoxical embolism.

Given the high specificity of cardiac CTA observed in our study, we propose enhancements to the diagnostic and treatment algorithm for PFO-associated stroke (Figure 5). After excluding common etiologies in stroke patients, those exhibiting a moderate to large shunt, as indicated by cTCD, and suspected of having a PFO-associated stroke, may benefit from cardiac CTA. Patients with a CTA-confirmed PFO may proceed directly to closure therapy, whereas those with negative CTA findings should undergo further evaluation with TEE. This modality can identify PFO and other potential cardiac sources of embolism. This diagnostic algorithm streamlines clinical workflows and minimizes unnecessary invasive procedures.

### Limitations

There are some limitations. First, this is a small-scale retrospective analysis, and some patients lacked original imaging data, which restricted the concurrent assessment of additional anatomical features such as PFO length and atrial septum secundum thickness. Second, cardiac CTA images were reconstructed using only mid-diastolic and end-systole phase, which precluded evaluation of the entire cardiac cycle. This imaging method potentially led to incomplete characterization of PFO dynamics and false-negative results. Third, the inability to perform the Valsalva maneuver during cardiac CTA limited the assessment of shunts through the PFO under standard imaging conditions. Future research should explore the integration of advanced techniques that enable the performance of Valsalva maneuver during cardiac CTA, thereby facilitating the evaluation of right-to-left shunts. In addition, our cohort was highly enriched with PFO-positive patients (prevalence 94.4%); selection bias may limit the generalizability of our findings. The specificity and negative predictive value cannot be reliably estimated due to the extremely low number of negative cases. Consequently, the reported diagnostic performance of cardiac CTA should not be generalized and must be interpreted with caution in unselected populations. Furthermore, the number of false-negative events in our cohort was relatively small (*n* = 31). While we included significant confounders in the multivariate model to adjust for bias, we acknowledge that the low events-per-variable ratio may lead to model overfitting and wider confidence intervals for some covariates. However, the primary association between shunt size and detection failure remained highly significant and robust. Finally, the image analysis in our study was performed by two radiologists using a consensus approach. Consequently, inter-observer variability was not strictly assessed, which may limit the reproducibility of our findings regarding subjective morphological features.

## 5. Conclusions

Cardiac CTA demonstrates high diagnostic value for PFO evaluation, particularly in patients with moderate-to-large RLS, where it shows superior accuracy compared to cases with small shunts. Therefore, in patients with suspected PFO-associated stroke who have positive preliminary screening results, cardiac CTA serves as a valuable tool for anatomical characterization and pre-procedural planning prior to transcatheter closure.

## Figures and Tables

**Table 1:** Diagnosis performance of cardiac CTA for PFO detection ^1^.

	All Individuals (*n* = 108)
Sensitivity (%)	70 (61–79)
Specificity (%)	100 (54–100)
Positive predictive value (%)	100 (95–100)
Negative predictive value (%)	16 (6–32)

^1^ Using transesophageal echocardiography as the reference standard. Abbreviations: PFO, patent foramen ovale; CTA, computer tomography angiography.

**Table 2:** Baseline clinical characteristics between two groups.

		Cardiac CTA for PFO		
	All Patients (*n* = 102)	CTA-Positive Group (*n* = 71)	CTA-Negative Group (*n* = 31)	*p* Value
Age, years	46.7 ± 14.9	44.0 ± 12.8	52.9 ± 17.5	0.005
Male, *n* (%)	47 (46.1)	27 (38.0)	20 (64.5)	0.014
BMI, kg/m^2^	24.3 ± 4.2	23.8 ± 3.4	25.5 ± 5.4	0.062
Heart rate, bpm	77.0 ± 11.4	76.8 ± 11.8	77.4 ± 12.9	0.828
SBP, mmHg	128.7 ± 15.8	127.5 ± 13.2	131.3 ± 20.4	0.270
DBP, mmHg	77.7 ± 11.4	76.6 ± 10.5	80.2 ± 13.2	0.147
Hypertension, *n* (%)	35 (34.3)	18 (25.4)	17 (54.8)	0.004
Diabetes mellitus, *n* (%)	14 (13.7)	6 (8.5)	8 (25.8)	0.019
Dyslipidemia, *n* (%)	10 (9.8)	6 (8.5)	4 (12.9)	0.487
Coronary artery disease, *n* (%)	8 (7.8)	1 (1.4)	7 (22.6)	<0.001
Smoking, *n* (%)	20 (19.6)	11 (15.5)	9 (29.0)	0.113
left atrial diameter, mm	34.8 ± 6.0	33.3 ± 4.9	38.3 ± 6.9	<0.001
RoPE score	5.6 ± 2.1	6.1 ± 1.8	4.5 ± 2.2	<0.001
PASCAL score, *n* (%)				<0.001
Unlikely	33 (32.4)	12 (16.9)	21 (67.7)	
Possible	38 (37.3)	31 (43.7)	7 (22.6)	
Probable	31 (30.4)	28 (39.4)	3 (9.7)	

Abbreviations: BMI, body mass index; SBP, systolic blood pressure; DBP, diastolic blood pressure; PASCAL, patent foramen ovale-associated stroke causal likelihood; RoPE, risk of paradoxical embolism, PFO, patent foramen ovale.

**Table 3:** Anatomy characteristics of PFO between two groups.

		Cardiac CTA for PFO		
	All Patients (*n* = 102)	CTA-Positive Group (*n* = 71)	CTA-Negative Group (*n* = 31)	*p* Value
PFO diameter, mm	2.10 ± 0.36	2.15 ± 0.35	1.97 ± 0.33	0.021
Atrial septal aneurysm, *n* (%)	23 (22.5)	18 (25.4)	5 (16.1)	0.305
Eustachian valve, *n* (%)	14 (13.7)	10 (14.1)	4 (12.9)	0.875
Atrial septal defect, *n* (%)	1 (1.0)	0 (0)	1 (3.2)	0.304
Degree of RLS on TEE				<0.001
Small, *n* (%)	46 (45.1)	23 (32.4)	23 (74.2)	
Moderate to large *, *n* (%)	56 (54.9)	48 (67.6)	8 (25.8)	
Degree of RLS on cTCD				<0.001
Small, *n* (%)	31 (30.4)	6 (19.4)	25 (80.6)	
Moderate to large *, *n* (%)	71 (69.6)	55 (77.5)	16 (22.5)	

* Moderate to large degree of RLS: more than 11 microbubbles. Abbreviations: RLS, right-to-left shunt; cTCD, contrast-enhanced transcranial Doppler; TEE, transesophageal echocardiography; PFO, patent foramen ovale.

**Table 4:** Univariate and multivariate analysis of factors associated with CTA false-negative results for PFO detection.

	Univariate Analysis			Multivariable Analysis		
	OR	95% CI	*p* Value	OR	95% CI	*p* Value
Age (per year)	1.044	1.012–1.076	0.007	0.982	0.933–1.034	0.493
Male	0.338	0.140–0.812	0.015	0.474	0.149–1.505	0.205
Hypertension	3.575	1.473–8.678	0.005	1.686	0.451–6.307	0.438
Coronary artery disease	20.417	2.388–174.569	0.006	6.176	0.581–65.666	0.131
Left atrial diameters (per mm)	1.164	1.068–1.269	<0.001	1.117	0.976–1.278	0.108
Moderate to large RLS *	0.070	0.024–0.200	<0.001	0.113	0.035–0.365	<0.001

* Evaluated by contrast-enhanced transcranial Doppler. Abbreviations: PFO, patent foramen ovale; RLS, right-to-left shunt; CTA, computed tomography angiography.

**Table 5:** Diagnosis sensitivities of detecting PFO at cardiac CTA in subgroup analysis *.

Subgroup		Patients Number	Sensitivity (95% CI)
Right-to-left shunt	Moderate to large	61	90.16% (82.69–97.64%)
	Small	47	39.02% (24.09–53.96%)
PFO diameter	≥2 mm	47	80.85% (69.60–92.10%)
	<2 mm	54	59.26% (46.15–72.36%)
Age	≥50	45	56.1% (40.91–71.29%)
	<50	63	78.69% (68.41–88.97%)
Sex	Male	51	59.57% (45.54–73.60%)
	Female	57	78.18% (67.27–89.10%)
Hypertension	Yes	40	54.29% (37.78–70.79%)
	No	68	77.61% (67.63–87.59%)
Coronary artery disease	Yes	9	12.5% (0–35.42%)
	No	99	74.47% (65.65–83.28%)

* Using transesophageal echocardiography as the reference standard. Abbreviations: PFO, patent foramen ovale; CTA, computed tomography angiography.

## Data Availability

The data that support the findings of this study are available from the corresponding author upon reasonable request.

## Abbreviations

The following abbreviations are used in this manuscript:

CTA	Computed Tomographic Angiography
PFO	Patent foramen ovale
TEE	Transesophageal echocardiography
TTE	Transthoracic echocardiography
cTCD	Contrast-enhanced transcranial Doppler
RLS	Right-to-left shunt
CLA	Channel-like appearance
CAD	Coronary artery disease
PASCAL	PFO-Associated Stroke Causal Likelihood
RoPE	Risk of paradoxical embolism
